# Overexpression of PD-1 on T cells promotes tolerance in cardiac transplantation via ICOS-dependent mechanisms

**DOI:** 10.1172/jci.insight.142909

**Published:** 2021-12-22

**Authors:** Thiago J. Borges, Naoka Murakami, Isadora T. Lape, Rodrigo B. Gassen, Kaifeng Liu, Songjie Cai, Joe Daccache, Kassem Safa, Tetsunosuke Shimizu, Shunsuke Ohori, Alison M. Paterson, Paolo Cravedi, Jamil Azzi, Peter T. Sage, Arlene H. Sharpe, Xian C. Li, Leonardo V. Riella

**Affiliations:** 1 Center for Transplantation Sciences, Department of Surgery, Massachusetts General Hospital, Harvard Medical School, Boston, Massachusetts, USA.; 2 Transplantation Research Center, Brigham and Women’s Hospital, Boston, Massachusetts, USA.; 3 Division of Nephrology, Massachusetts General Hospital, Harvard Medical School, Boston, Massachusetts, USA.; 4 Department of Immunology, Blavatnik Institute, Harvard Medical School, Boston, Massachusetts, USA.; 5 Evergrande Center for Immunologic Diseases and Department of Pathology, Harvard Medical School, Brigham and Women’s Hospital, Boston, Massachusetts, USA.; 6 Renal Division, Department of Medicine, Icahn School of Medicine at Mount Sinai, New York, New York, USA.; 7 Immunobiology and Transplant Science Center, Houston Methodist Hospital, Houston, Texas, USA.

**Keywords:** Immunology, Transplantation, Costimulation, T cells, Tolerance

## Abstract

The programmed death 1/programmed death ligand 1 (PD-1/PD-L1) pathway is a potent inhibitory pathway involved in immune regulation and is a potential therapeutic target in transplantation. In this study, we show that overexpression of PD-1 on T cells (PD-1 Tg) promotes allograft tolerance in a fully MHC-mismatched cardiac transplant model when combined with costimulation blockade with CTLA-4–Ig. PD-1 overexpression on T cells also protected against chronic rejection in a single MHC II–mismatched cardiac transplant model, whereas the overexpression still allowed the generation of an effective immune response against an influenza A virus. Notably, Tregs from PD-1 Tg mice were required for tolerance induction and presented greater ICOS expression than those from WT mice. The survival benefit of PD-1 Tg recipients required ICOS signaling and donor PD-L1 expression. These results indicate that modulation of PD-1 expression, in combination with a costimulation blockade, is a promising therapeutic target to promote transplant tolerance.

## Graphical abstract

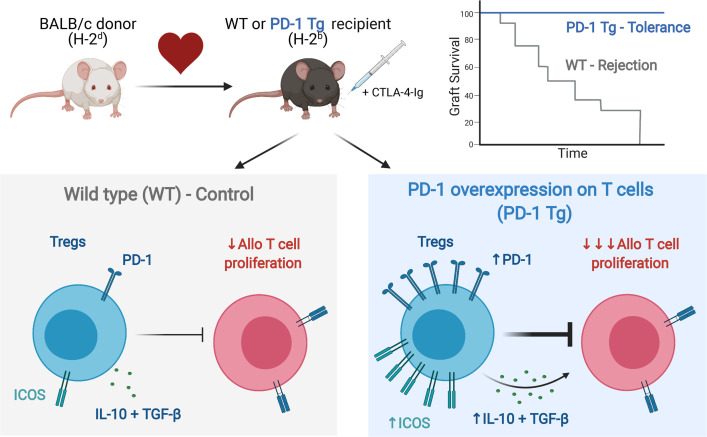

## Introduction

Programmed death 1 (PD-1) receptor plays a major inhibitory role in T cell activation via interaction with its ligands PD-L1 and PD-L2, resulting in decreased T cell activation, proliferation, differentiation, and cytokine production (1). PD-1 is upregulated by T cells after antigen-mediated activation of the T cell receptor (TCR), and its expression is decreased once the antigen is cleared (2, 3). Chronic stimulation of T cells by the antigen leads to high and continuous expression of PD-1, generating an exhausted T cell phenotype (4). PD-1 is expressed on thymocytes and all CD4^+^ and CD8^+^ T cells, including Tregs, exhausted T cells, and memory T cells (5). PD-1 can also be expressed by B cells, NK cells, NKTs, some myeloid cells, and cancer cells (5). In an attempt to induce immune regulation in transplantation, the PD-1/PD-L1 pathway has become an attractive target. PD-1 signaling is critical to protect against rejection in mouse heart (6–9), liver (10), and skin transplant models (11), but the consequence of PD-1 overexpression on T cells is unknown.

In this study, we investigated whether overexpression of PD-1 on T cells could promote long-term graft survival. Using PD-1 Tg mice (12), we show that T cell–specific overexpression of PD-1 promoted tolerance in a fully MHC-mismatched murine cardiac transplant model in combination with a single administration of costimulation blockade. Mechanistically, PD-1 Tg conventional T cells (Tconv; CD4^+^Foxp3^–^) proliferated significantly less and produced fewer proinflammatory cytokines compared with WT Tconv. Also, graft-infiltrating PD-1 Tg Tregs expressed significantly higher levels of ICOS. In vivo blockade of ICOS prevented tolerance of PD-1 Tg recipients. Using PD-L1–KO donor allografts, we demonstrated that allograft PD-L1 expression was required for this survival benefit. These results suggest that the modulation of the PD-1/PD-L1 axis could be a promising strategy to induce tolerance in organ transplantation.

## Results

### PD-1 is highly expressed on T cells of PD-1 Tg mice.

**Figure 1:**
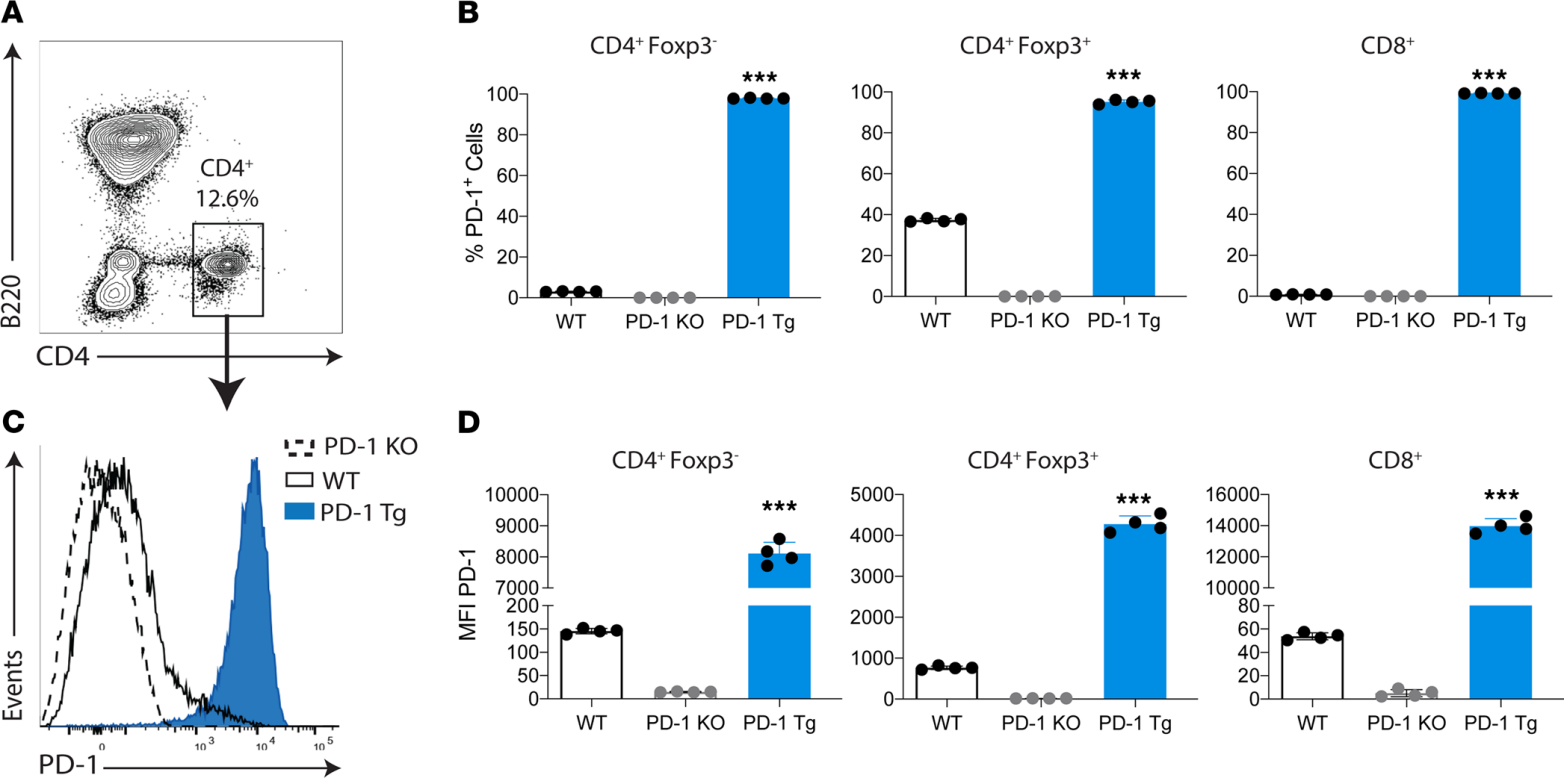
T cell–specific PD-1 overexpression in PD-1 Tg mice. (**A** and **B**) Percentage of PD-1**–**expressing CD4^+^Foxp3^–^, CD4^+^Foxp3^+^, and CD8^+^ T cells of WT, PD-1–KO, and PD-1 Tg mice. (**C**) Representative histograms of PD-1 expression on Tregs and (**D**) PD-1 MFI on CD4^+^ Tconv, Tregs, and CD8^+^ T cells of WT, PD-1–KO, and PD-1 Tg mice. *n* = 4 mice/group. Representative data of 5 independent experiments are presented. For all panels, the bar graphs represent mean ± SD. ***P < 0.001 (1-way ANOVA with Bonferroni post hoc test).

We initially analyzed the expression of PD-1 on splenocytes from unmanipulated B6 WT, PD-1–KO, and PD-1 Tg mice (Figure 1). We confirmed that PD-1 Tg mice overexpressed PD-1 on splenic T-cells, as previously described (12). The percentage (Figure 1, A and B) and expression level (Figure 1, C and D) of PD-1 on T cells were significantly higher in PD-1 Tg mice compared with WT CD4^+^ T cells, CD8^+^ T cells, and Tregs; this higher expression of PD-1 in PD-1 Tg mice was not observed on B cells, macrophages, or DCs (data not shown). Because PD-1 is expressed by thymocytes and may participate in T cell development by inhibiting positive selection (13), we characterized the thymocytes of PD-1 Tg mice and did not observe significant differences in the proportion of double-negative (CD4^–^CD8^–^), double-positive (CD4^+^CD8^+^), and single-positive (CD4^+^CD8^–^ or CD4^–^CD8^+^) T cells when compared with WT controls (Supplemental Figure 1; supplemental material available online with this article; https://doi.org/10.1172/jci.insight.142909DS1).

### PD-1 Tg mice achieved tolerance in mouse models of acute and chronic cardiac transplant rejection.

**Figure 2:**
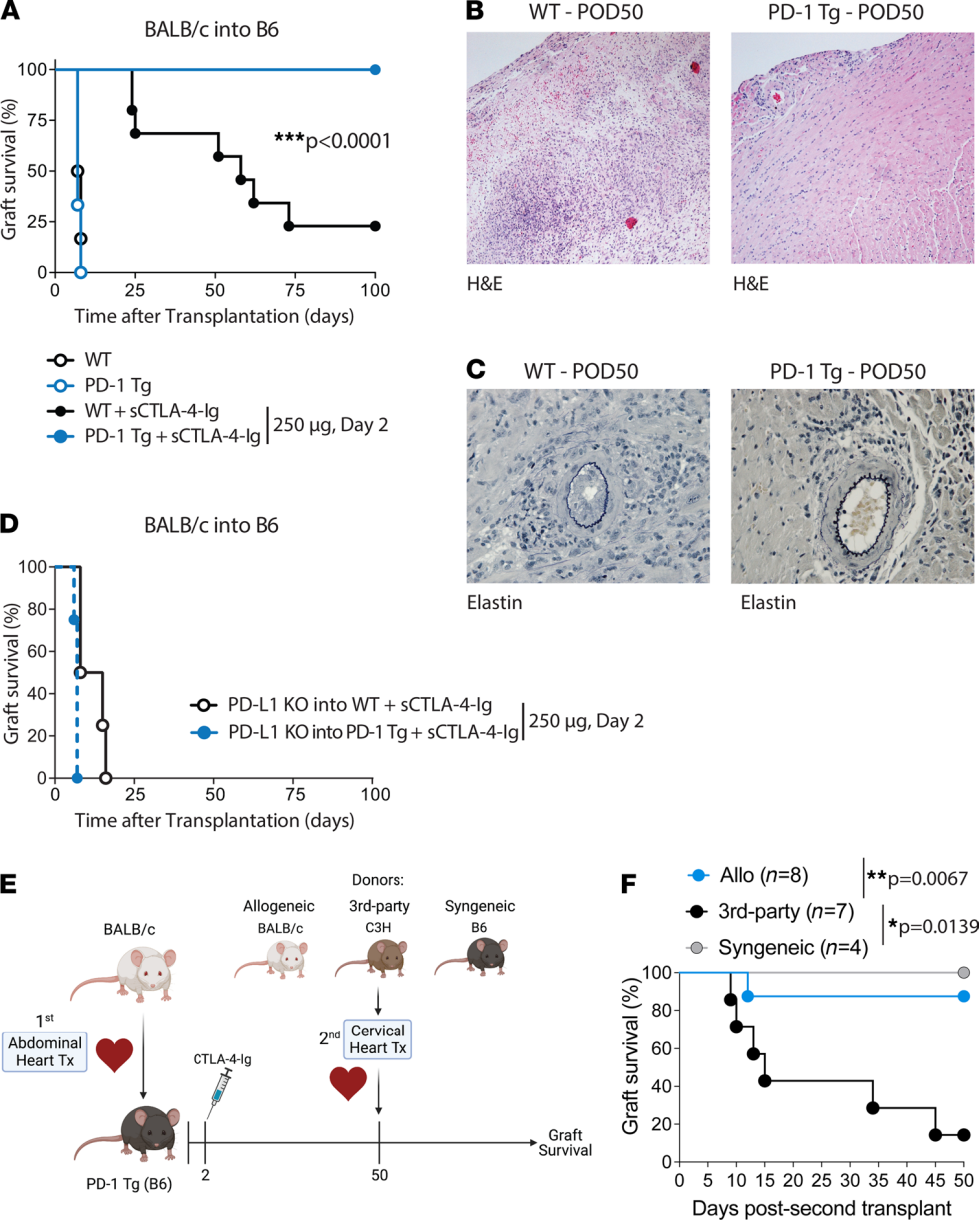
A single dose of CTLA-4–Ig induced tolerance in PD-1 Tg recipient and depends on PD-L1 expression by donor cells. (**A**) Kaplan-Meier curves of allograft survival of fully MHC-mismatched cardiac transplants; BALB/c hearts were transplanted into WT or PD-1 Tg C57BL/6 recipients with or without a single dose of CTLA-4–Ig (250 μg on day 2 after transplant). In the presence of CTLA-4–Ig, allograft survival was significantly prolonged in the PD-1 Tg group, according to log-rank test, when compared with the WT group (*n* = 6/group). Representative histology of cardiac allografts retrieved from WT or PD-1 Tg mice (treated with sCTLA-4–Ig on day 2) on day 50 after transplant and stained with (**B**) H&E (magnification, ×20) and (**C**) elastin (magnification, ×40). PD-1 Tg recipients had a normal tissue architecture, minimal lymphocyte infiltration. and minimal intimal proliferation (*n* = 6/group; magnification, ×40). (**D**) Kaplan-Meier curves of allograft survival of PD-L1–KO hearts (BALB/c hearts) transplanted into WT or PD-1 Tg C57BL/6 mice with a single dose of 250 μg of CTLA-4–Ig on day 2. (**E**) Schematic experimental design of abdominal and cervical heart cotransplantation in PD-1 Tg mice. PD-1 Tg recipients (H-2^b^) were transplanted with abdominal BALB/c hearts (H-2^d^) and treated with 250 μg of CTLA-4–Ig at day 2 after transplant. Fifty days later, the same animals were cervically implanted with BALB/c (H-2^d^; allo), C3H (H-2^k^; third-party) or B6 (H-2^b^; syngeneic) hearts (*n* = 4–8/group). The grafts were assessed by monitoring palpitation for the following 50 days. Cartoon created with BioRender.com. (**F**) Kaplan-Meier survival curves of the second cardiac grafts for each group. *P* values determined by log-rank test. Allo, allogeneic; POD, postoperative day; Tx, transplant. *P < 0.05; **P < 0.01; ***P < 0.001.

To test the hypothesis that PD-1 overexpression prolongs allograft survival, BALB/c (H-2^d^) mouse hearts were transplanted into WT or PD-1 Tg B6 (H-2^b^) mice with or without a single dose of costimulation blockade with CTLA-4–Ig. Rejection tempo was similar in WT and PD-1 Tg recipients not treated with CTLA-4–Ig, with a median survival time (MST) of 7 days. Conversely, treating PD-1 Tg recipients with a single dose of CTLA-4–Ig on day 2 resulted in long-term allograft survival (MST, >100 days) compared with an MST of 56 days in WT recipients (Figure 2A). Histopathology of the tolerized allografts revealed minimal inflammation, low fibrosis percentage (<3%), and vasculopathy score (<1) at 50 days (Figure 2, B and C) or 100 days (Supplemental Figure 2) after transplantation. In sum, overexpression of PD-1 on T cells improved allograft survival in the presence of early costimulation blockade.

Given this benefit in the acute rejection model of BALB/c into B6 transplantation, we evaluated the impact of high PD-1 expression on a single MHC II–mismatched cardiac transplant model (bm12 into B6). This model has been previously described to result in chronic allograft vasculopathy (14–16). Most allografts survived long term, except for 1 of the grafts in WT recipients that was rejected at day 50 after transplant (*P* = 0.37) (Supplemental Figure 3A). At 8 weeks after transplant, we compared bm12 allograft histopathology between WT and PD-1 Tg recipients, which revealed significantly lower International Society of Heart and Lung Transplantation (ISHLT) rejection grades, myocyte loss, fibrosis, chronic rejection, and vasculitis in cardiac allografts transplanted into the PD-1 Tg mice compared with WT recipients (Supplemental Figure 3, B and C). This suggests that PD-1 overexpression on T cells is protective of chronic rejection in this MHC II–mismatched transplant model.

### PD-L1 expression in donor allograft is required for survival advantage in PD-1 Tg mice.

PD-1 transduces signals via its interaction with PD-L1 and PD-L2. To determine whether PD-L1 expression on cardiac donor allograft is required for the increased survival observed in PD-1 Tg recipients, we transplanted hearts from PD-L1–KO mice on the BALB/c background into either PD-1 Tg or WT B6 mice and treated them with a single dose of CTLA-4–Ig on day 2. The survival benefit was completely abrogated in the absence of PD-L1 on the graft (MST, 7 days compared with >100 days; Figure 2D), indicating that PD-L1–PD-1 interaction in the allograft is necessary for PD-1 Tg T cells to promote long-term graft survival.

### Long-term allograft survival in PD-1 Tg mice is donor specific.

We next evaluated whether the long-term cardiac graft survival achieved in PD-1 Tg mice was antigen-specific. To determine that, we transplanted PD-1 Tg mice with abdominal BALB/c hearts and treated the animals with a single dose of CTLA-4–Ig on day 2. Fifty days later, the same animals were cotransplanted with cervical BALB/c (allogeneic), C3H (third-party) or B6 (syngeneic) hearts (Figure 2E). Cotransplantation of a second BALB/c heart into PD-1 Tg recipients resulted in long-term acceptance of 9 out of 10 cardiac grafts (90%), as assessed by monitoring palpitation (MST, >50 days; Figure 2F). In contrast, third-party (H-2^k^) hearts cotransplanted into PD-1 Tg recipients showed an accelerated graft rejection compared with those from allo-BALB/c and B6 syngeneic donors (MST, 15 days compared with >50 days; Figure 2E). None of the first abdominal BALB/c hearts were rejected. Our data indicate that PD-1 Tg mice treated with early costimulation blockade have a donor-specific long-term immune tolerance.

### PD-1 Tg effector T cells are less primed and proliferate significantly less than WT T cells in vitro and in vivo.

To evaluate the cellular mechanisms behind the improved allograft survival in PD-1 Tg mice, we analyzed splenocytes from WT or PD-1 Tg recipients of BALB/c hearts treated with a single dose of CTLA-4–Ig, 3 weeks after transplantation (which was the time of initiation of graft rejection in WT recipients). There was no significant difference in CD4^+^ and CD8^+^ effector memory (CD44^+^CD62L^lo^) T cell percentages and numbers in the spleens of transplanted mice of WT or PD-1 Tg recipients (mean, 39.7% versus 46.4% in the CD4^+^ WT group, *P* = 0.410; mean 30.5% versus 35.1% in the CD8^+^ WT group, *P* = 0.349).

**Figure 3:**
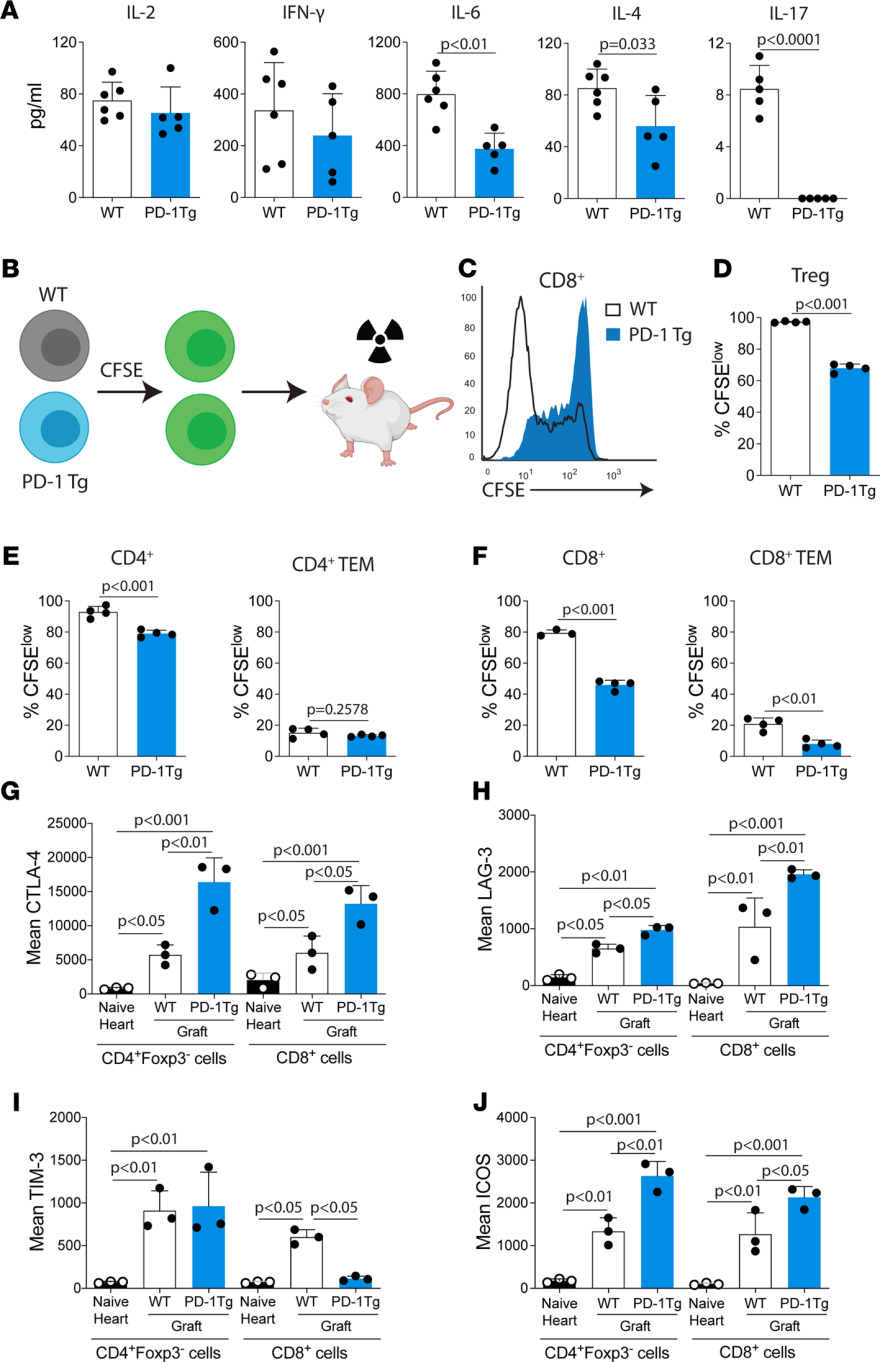
PD-1 Tg T cells are less primed and proliferate significantly less in vivo in response to allostimulation. (**A**) WT or PD-1 Tg splenocytes were harvested at 14 days after cardiac allograft transplants and 1 dose of CTLA-4–Ig and then cocultured with allogeneic, irradiated, donor-type stimulator cells for 72 hours. Cytokine production in the culture supernatant was measured using the Luminex assay. *n* = 5–6 mice/group. *P* values determined by *t* test. Data are shown from 1 representative experiment of 3 independent experiments. (**B**) Modified graft-versus-host disease (GVHD) model in which WT or PD-1 Tg splenocytes were labeled with CFSE and adoptively transferred into sublethally irradiated BALB/c mice. Cartoon created with BioRender.com. (**C**) Representative CFSE histograms of CD8^+^ cells by flow cytometry 72 hours after adoptive transfer (lower CFSE signal is a marker of greater cell proliferation). PD-1 Tg compared with WT percentages of (**D**) CFSE^lo^ Tregs, (**E**) CD4^+^, and (**F**) CD8^+^ T cells. *n* = 4 animals/group. *P* values in **D** determined by *t* test. Expression of (**G**) CTLA-4, (**H**) LAG-3, (**I**) TIM-3, and (**J**) ICOS in graft-infiltrating CD4^+^ Tconv (defined as CD4^+^Foxp3^–^ T cells) and CD8^+^ T cells from WT or PD-1 Tg recipients on day 7 after transplant (1 dose of CTLA4-Ig on day 2). *n* = 3 animals/group. *P* values determined by 1-way ANOVA with Tukey post hoc test. Data are representative of 4 independent experiments. For all panels, the bar graphs represent mean ± SD. TEM, T effector memory cells.

To further investigate the effector T cell functions of PD-1 Tg recipients, ex vivo cytokine production upon mixed lymphocyte reaction was measured using the Luminex system. When stimulated with donor-derived irradiated splenocytes, PD-1 Tg splenocytes from heart-transplanted mice given CTLA-4–Ig produced significantly less IL-4, IL-6, and IL-17 compared with WT mice (Figure 3A). Furthermore, there was a slight reduction in IFN-γ and IL-2 secretion by PD-1 Tg splenocytes, although the reduction did not reach significance. Thus, our data suggest that following heart transplantation, T effector cells from PD-1 Tg mice are less functional than T effector cells from WT hosts.

To further assess the cell-intrinsic inhibitory phenotype of PD-1–overexpressing T cells in vivo, we used a graft-versus-host disease (GVHD) model that allows us to track and measure polyclonal, alloreactive T cell proliferation and activation in vivo after adoptive transfer into an allogeneic recipient (17). PD-1 Tg or WT splenocytes were labeled with CFSE and adoptively transferred into sublethally irradiated BALB/c mice (Figure 3B). Seventy-two hours after the transfer, the proliferation of transferred T cells was assessed by CFSE dilution (Figure 3C). When compared with WT, PD-1 Tg Tregs (Figure 3D), CD4^+^ cells (Figure 3E), and CD8^+^ cells (Figure 3F) proliferated significantly less. The analysis of T effector memory cell subsets demonstrated that proliferation of CD4^+^ effector memory cells was similar between the groups (Figure 3E), but CD8^+^ effector memory cell proliferation was significantly decreased in the PD-1 Tg group (Figure 3F). In PD-1 Tg animals, CD8^+^ T cells had a higher mean expression of surface PD-1 when compared with CD4^+^ T cells (MFI of surface PD-1 on CD8^+^ and CD4^+^ T cells, respectively: 13,972 ± 477 versus 8108 ± 364, *P <* 0.0001; Figure 1D). This finding suggests that PD-1 could have a stronger cell-intrinsic inhibitory phenotype in CD8^+^ T cells. Overall, the data suggest that PD-1 overexpression impairs T cell proliferation both in vitro and in vivo.

PD-1 upregulation is known to be a marker for exhausted T cells along with other coinhibitory molecules such as TIM-3, LAG-3, and CTLA-4 (18). Although graft-infiltrating Tconv and CD8^+^ T cells from PD-1 Tg recipients expressed more CTLA-4 (Figure 3G) and LAG-3 (Figure 3H) when compared with naive hearts or WT recipients, the expression of other receptors, like TIM-3, was unchanged or decreased (Figure 3I), and ICOS was increased (Figure 3J) at 7 days after transplant. These data suggest that T cells from PD-1 Tg mice may have a partially exhausted phenotype.

To determine whether PD-1 Tg cells had diminished effector responses in settings other than solid organ transplantation, we used an influenza infection model, because the PD-1 pathway has important roles in viral infection. We infected naive WT or PD-1 Tg animals with a sublethal dose of influenza virus (strain A/Puerto Rico/8/1934 H1N1). PD-1 Tg animals were equally capable of controlling viral infection as WT controls, as determined by weight loss (Supplemental Figure 4A) and survival (Supplemental Figure 4B). Also, there was no difference in viral loads analyzed at day 7 after infection (Supplemental Figure 4C). Our data demonstrate that PD-1 Tg mice can mount an optimal immunologic response to acute viral infection despite the high PD-1 expression on T cells.

### Tregs are fewer in PD-1 Tg recipients but express higher amounts of IL-10 and latency-associated peptide.

**Figure 4:**
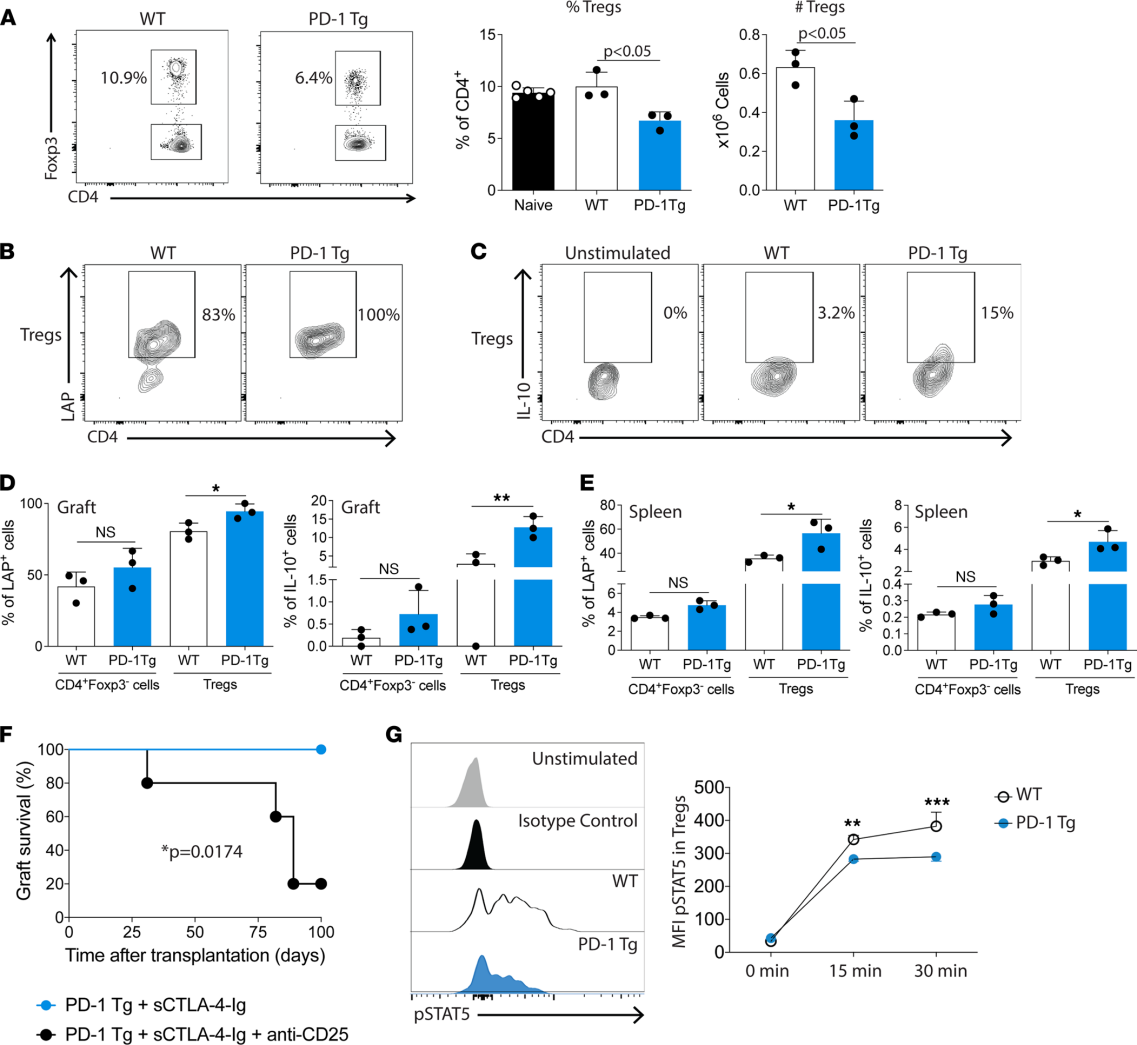
Comparison of WT and PD-1 Tg Tregs in allogeneic transplant recipients. WT or PD-1 Tg recipients of fully MHC-mismatched cardiac transplantation received a single dose of CTLA-4–Ig (250 μg/mouse, i.p.) on day 2 after transplantation. (**A**) Seven days after heart transplants, WT and PD-1 Tg splenocyte populations were analyzed by flow cytometry. Tregs (CD4^+^Foxp3^+^ cells) were significantly reduced in the PD-1 Tg group (*n* = 3–5/group) according to 1-way ANOVA with Tukey post hoc test. Data are representative of 4 independent experiments. (**B**) Representative contour plots of LAP expression and (**C**) IL-10 production by WT or PD-1 Tg graft-infiltrating Tregs (CD4^+^Foxp3^+^ cells). Percentage of LAP^+^ or IL-10^+^ (**D**) infiltrating or (**E**) splenic Tconv (CD4^+^Foxp3^–^ cells) and Tregs from WT or PD-1 Tg heart recipients. Data are representative of 2 independent experiments (*n* = 3 animals/group). Data were analyzed using a 1-way ANOVA with Tukey post hoc test. *P < 0.05, **P < 0.01. For all panels, the bar graphs represent mean ± SD. (**F**) Allograft survival of BALB/c heart allografts transplanted to PD-1 Tg C57BL/6 mice treated with a single dose of CTLA-4–Ig (250 μg on day 2 after transplant). One group received 100 μg of anti-CD25 Ab on days –7 and –1, which impaired the survival induced by sCTLA-Ig treatment (*n* = 5 per group; *P = 0.0174). (**G**) Expression of phosphorylated-STAT5 (pSTAT5) in WT and PD-1 Tg Tregs stimulated with IL-2 (5 ng/mL) for 15 or 30 minutes to induce the expression of pSTAT5 (MFI was obtained by flow cytometry). Experiments were performed in triplicate with a pool of 3 mice and were analyzed using a 2-way ANOVA with Sidak post hoc test. **P < 0.01, ***P < 0.001 versus PD-1 Tg mice.

Tregs are instrumental for the induction and maintenance of tolerance to auto- and alloantigens (19). To investigate whether Tregs were involved in the tolerance mechanism of PD-1 Tg mice, we analyzed Tregs in the spleen of WT and PD-1 Tg transplanted animals at day 7 after transplant. Treg percentages and counts (CD4^+^Foxp3^+^) were significantly lower in PD-1 Tg recipients compared with WT (Figure 4A). Naive PD-1 Tg animals also had a reduced percentage and absolute numbers of Tregs in the spleen and thymus compared with WT controls (Supplemental Figure 5).

We next hypothesized that PD-1 overexpression could be affecting the suppressive function of the Tregs. Graft-infiltrating Tconv and Tregs from WT or PD-1 Tg recipients of fully MHC-mismatched cardiac transplantation receiving CTLA4-Ig were analyzed for the production of IL-10 and the expression of membrane-bound TGF-β1 through the analysis of the latency-associated peptide (LAP). Indeed, Tregs from PD-1 Tg recipients had higher expression of LAP (Figure 4, B, D, and E) and increased production of IL-10 (Figure 4, C–E) when compared with WT cells. Furthermore, there was a slight increase in LAP expression and IL-10 secretion by PD-1 Tg Tconv, although it did not reach significance (Figure 4, B–E). Last, depletion of Tregs using anti-CD25 Ab before transplantation shortened graft survival of PD-1 Tg recipients treated with costimulation blockade (MST, 89 days versus > 100 days, *P* = 0.0174; Figure 4F), indicating the important role of Tregs for long-term graft acceptance in PD-1 Tg recipients.

mTOR (20) and STAT5 signaling pathways are crucial for Treg function (21). Therefore, we next investigated whether they were responsible for the greater regulatory effects of PD-1 overexpression on T cells. We hypothesized that PD-1 overexpression could inhibit the mTOR pathway, thereby enhancing Treg function. We treated naive WT or PD-1 Tg T cells with PMA/ionomycin at different time points and analyzed the phosphorylation levels of S6K-1 (pS6K), a known mTOR downstream target (22). However, no differences were found in the levels of pS6K between PD-1 Tg and WT Treg cells, suggesting that the mTOR pathway is not affected by PD-1 overexpression (Supplemental Figure 6).

We next investigated the phosphorylation level of STAT5, a transcription factor downstream of the IL-2 signaling pathway that binds to the *Foxp3* promoter and supports Treg development (23). We treated WT or PD-1 Tg Tregs with 5 ng/mL recombinant murine IL-2 for 15 or 30 minutes and analyzed the expression of pSTAT5 by flow cytometry. In the presence of IL-2, PD-1–overexpressing Tregs had less phosphorylation of STAT5 than did WT Tregs (Figure 4G).

In summary, Tregs are required for allograft tolerance in PD-1 Tg recipients and exhibit an increased expression of IL-10 and LAP, despite less Treg proliferation. This effect was not mediated by an increase in STAT5 activation or mTOR inhibition.

### ICOS is upregulated in PD-1 Tg Tregs.

**Figure 5:**
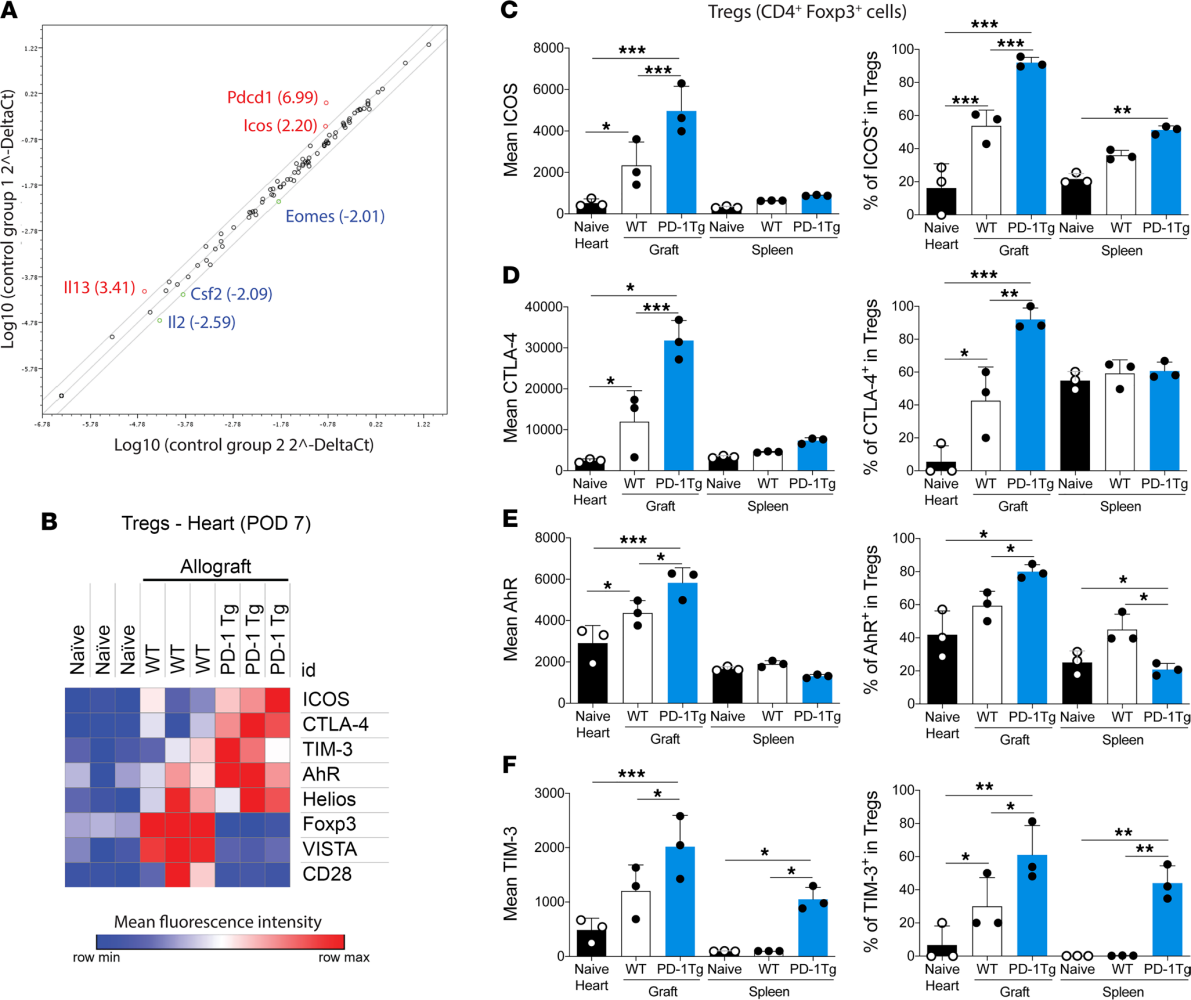
ICOS is upregulated in Tregs from PD-1 Tg recipients treated with CTLA-4–Ig. (**A**) Volcano plot representing differential expressed genes in flow-sorted PD-1 Tg versus WT Tregs from naive animals. (**B**) Heatmap representing the expression of costimulatory and coinhibitory molecules by graft-infiltrating Tregs on day 7 after transplant. Mean expression (left panel) of (**C**) ICOS, (**D**) CTLA-4, (**E**) AhR, and (**F**) TIM-3 by Tregs, and percentages of (**C**) ICOS^+^, (**D**) CTLA-4^+^, (**E**) AhR^+^, and (**F**) TIM-3^+^ Tregs isolated from native hearts, cardiac allografts, or spleens on day 7 after transplant. (**C–F**) *n* = 4–5 mice per group. *P < 0.05, **P < 0.01, ***P < 0.001, by *t* test with multiple testing correction using the Holm-Sidak method; α = 0.05, n = 3 *t* tests. For all panels, the bar graphs represent mean ± SD. POD, postoperative day.

Because PD-1 overexpression did not change the levels of mTOR and decreased pSTAT5, we further investigated PD-1 Tg Tregs, using an reverse transcription PCR array of sorted WT or PD-1 Tg Tregs (CD4^+^Foxp3^+^ cells) from naive animals. The expression of *Pdcd1*, *Icos*, and *Il13* were significantly upregulated, whereas *Eomes*, *Csf2*, and *Il2* were downregulated in PD-1 Tg Tregs, compared with WT cells (Figure 5A).

We performed an extensive immunophenotyping of Tregs from naive hearts, cardiac grafts, and spleens from WT or PD-1 Tg recipients 7 days after transplant. Immunophenotypic characterization of the graft-infiltrating Tregs showed significantly higher expression of ICOS, CTLA-4, AhR, and TIM-3 (Figure 5, B–F). TIM-3 was also upregulated in splenic Tregs (Figure 5F). In addition to higher expression levels (determined by MFI), we also found an increase in the percentages of ICOS^+^, CTLA-4^+^, AhR^+^, and TIM-3^+^ intragraft Tregs in PD-1 Tg recipients. In the spleen of PD-1 Tg animals, the proportions of ICOS^+^ (Figure 5C) and TIM-3^+^ (Figure 5F) Tregs were increased, whereas the proportion of AhR^+^ Tregs was decreased (Figure 5E).

### ICOS is required for the induction of prolonged immune modulation in PD-1 Tg recipients.

**Figure 6:**
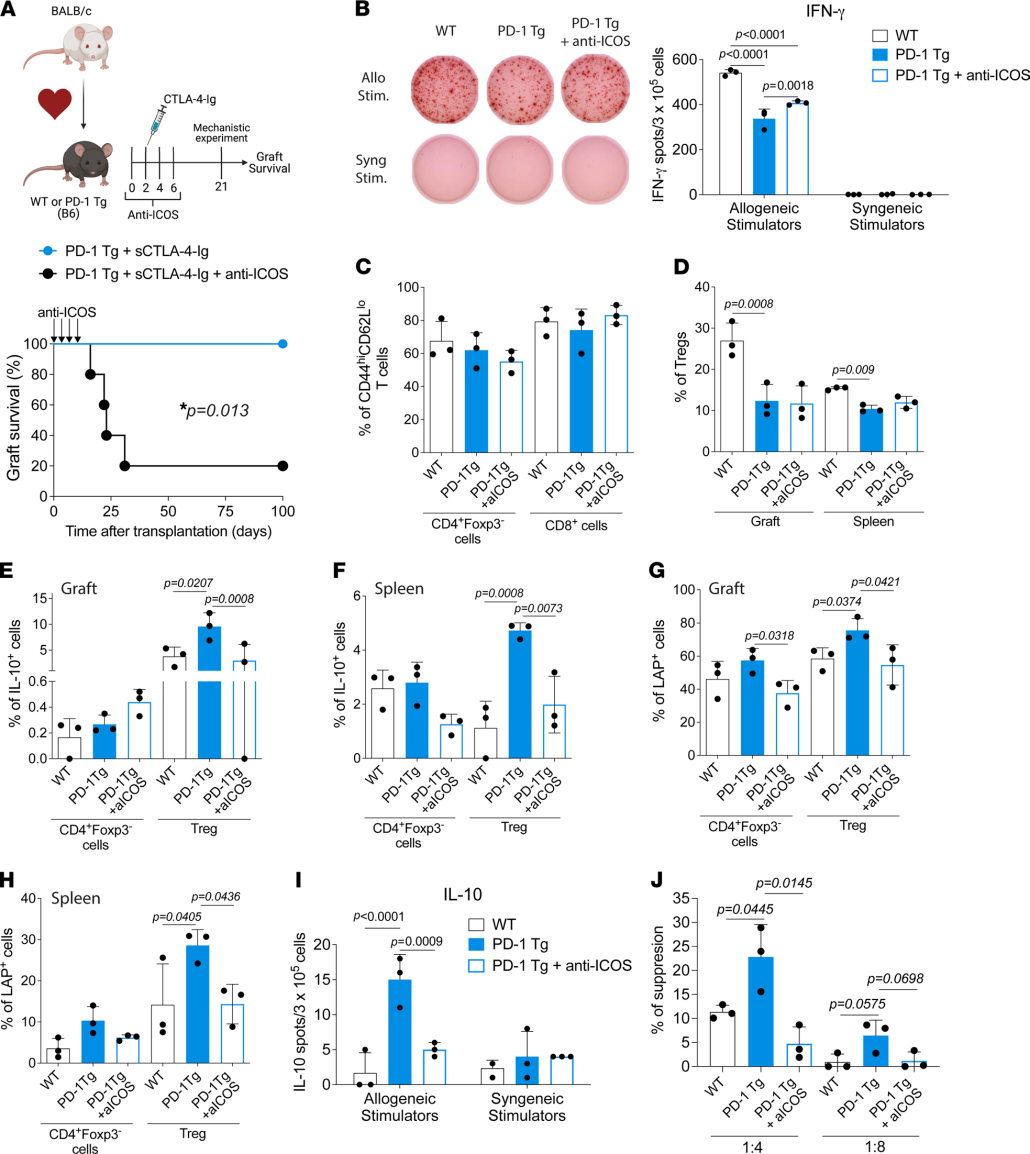
Induction of tolerance in PD-1 Tg recipients treated with CTLA-4–Ig is ICOS dependent. (**A**) Graft survival in PD-1 Tg recipients treated with a single dose of CTLA-4–Ig with or without transient ICOS blockade (days 0, 2, 4, and 6 after transplant). Rat IgG2b was used as an isotype control. Long-term survival of PD-1 Tg recipients was abolished upon the interruption of ICOS signaling. (*n* = 5 per group; *P < 0.013). (**B**) T cells from the spleen were magnetically isolated and cultured with allogeneic (allo; B6) or syngeneic (syng; BALB/c) irradiated splenocytes for 24 hours in anti-mouse IFN-γ–coated plates. IFN-γ production by T cells was measured by ELISPOT (number of spots per million T cells ± SD in triplicate). Statistics using 1-way ANOVA with Tukey post-test. Representative results of 2 independent experiments. (**C**–**H**) Twenty-one days after heart transplants, splenocytes and allograft populations from WT and PD-1 Tg animals and PD-1 Tg animals treated with anti-ICOS were analyzed by flow cytometry. (**C**) Frequency of intragraft effector memory cells (CD44^hi^CD62L^lo^) in Tconv (CD4^+^Foxp3^–^ cells) and CD8^+^ T cell populations at day 21 after transplant. (**D**) Frequency of graft-infiltrating and splenic Tregs (CD4^+^Foxp3^+^ cells). LAP expression and IL-10 production by (**E** and** G**) graft-infiltrating or (**F** and** H**) splenic Tconv (CD4^+^Foxp3^–^ cells) and Tregs from WT, PD-1 Tg controls, or PD-1 Tg mice treated with anti-ICOS. Data are representative of 2 independent experiments (*n* = 3 animals/group). Data were analyzed using 1-way ANOVA with Tukey post hoc test. (**I**) T cells from the spleen were magnetically isolated and cultured with allo (B6) or syng (BALB/c) irradiated splenocytes for 48 hours in anti-mouse IL-10–coated plates. IL-10 production by T cells was measured by ELISPOT (number of spots per million T cells ± SD in triplicate). Data were analyzed by 1-way ANOVA with Tukey post hoc test. Representative results of 2 independent experiments are presented. (**J**) Flow-sorted splenic CD4^+^ Foxp3-GFP^+^ Tregs were isolated from recipients as described in **A**, and incubated in varying ratios (i.e., 1:4, 1:8) ex vivo with CD4^+^Foxp3-GFP^–^ (Tconv) isolated from WT mice and stimulated with anti–CD3/CD28–conjugated beads for 72 hours. For all panels, the bar graphs represent mean ± SD.

Because ICOS was increased at both mRNA (Figure 5A) and protein (Figure 5C) levels, we assessed its potential role in vivo using a blocking Ab approach against ICOS at the time of transplant. We transplanted BALB/c hearts into PD-1 Tg recipients and treated tolerant recipients with the anti-ICOS Ab or isotype control on days 0, 2, 4, and 6 (Figure 6A). This ICOS early blockade regimen has been demonstrated to have minimal effects on the improvement of graft survival (24). Prolongation of allograft survival in PD-1 Tg recipients was abrogated upon ICOS blockade, indicating a critical role for ICOS in tolerance induction (Figure 6A).

We next investigated whether anti-ICOS therapy inhibited alloreactive T cell responses at day 21 after transplant by enzyme-linked immune absorbent spot (ELISPOT). Fewer IFN-γ–producing, splenic, anti-donor T cells from PD-1 Tg animals were observed when compared with those from WT recipients (Figure 6B). In PD-1 Tg recipients, anti-ICOS treatment slightly increased the number of IFN-γ–producing alloreactive T cells, but the number was still significantly lower than in WT controls (Figure 6B). ICOS blockade had no effect on CD4^+^ and CD8^+^ effector memory (CD44^hi^CD62^lo^) T cells (Figure 6C) or Treg frequency (Figure 6D). Thus, our data suggest that anti-ICOS treatment has minimal effects on effector T cell responses.

We hypothesized that ICOS blockade could be affecting the suppressive function of the PD-1 Tg Tregs. Indeed, both intragraft and splenic Tregs from PD-1 Tg recipients treated with anti-ICOS had decreased production of IL-10 (Figure 6, E and F) and LAP expression (Figure 6, G and H). Anti-ICOS treatment had no effects on the IL-10 production and LAP expression by non-Tregs (Figure 6, E–H). We verified by ELISPOT that the increased IL-10 production in PD-1 Tg recipients was allospecific, and it was inhibited by the ICOS blockade (Figure 6I).

Last, we observed that flow-sorted CD4^+^Foxp3-GFP^+^ Tregs from PD-1 Tg heart recipients had a superior ability to inhibit T cell proliferation in an in vitro suppression assay than did WT Tregs (Figure 6J). This effect was abrogated by the anti-ICOS treatment (Figure 6J). Altogether, our data demonstrated that the anti-ICOS treatment has a dominant inhibitory effect on Treg suppressive function.

## Discussion

In this work, we show in a mouse cardiac transplant model that the overexpression of PD-1 on T cells significantly prolongs allograft survival and protects against chronic rejection in the setting of early B7:CD28 blockade with CTLA-4–Ig. The importance of the PD-1/PD-L1 pathway in mediating alloimmune regulation has been investigated in several prior studies, most of which used either a blocking Ab or genetic deletion. For example, CTLA-4–Ig–induced tolerance was abrogated in PD-L1 KO recipients of BALB/c hearts or WT recipients treated with anti–PD-L1 blocking Abs (7). On the other hand, the use of PD-1 agonistic agents such as PD-L1-Ig in addition to cyclosporine or rapamycin resulted in prolonged allograft survival (6).

The requirement of the early blockade of the B7:CD28 signal to achieve the significant survival difference is most likely related to the competitive opposite signaling effects of PD-1 and CD28 on T cells (3). PD-1 transduces an inhibitory signal in part via the recruitment of phosphate SHP-2, which downregulates CD28-mediated PI3K activity (25). A strong CD28 signal can easily overcome PD-1 inhibition and simultaneous blockade of B7:CD28 with an enhanced PD-1 signal demonstrated synergism in regulating the alloimmune response. Another mechanism of PD-1 inhibition of T cells is by affecting the stability of the antigen-presenting cell–T cell (APC–T cell) synapse. Through intravital microscopy, PD-1 signaling was shown to block the TCR “stop signal” required for an effective and prolonged APC-T cell interaction and consequent T cell activation (26–28). Therefore, overexpression of PD-1 may promote transient and incomplete APC-T cell interactions, leading to a significant reduction in T cell activation and proliferation. Nonetheless, these PD-1 Tg T cells are still capable of mounting an effective immune response in the absence of costimulation blockade, as evident in both an acute murine cardiac transplantation model and a model of influenza A infection.

A unique feature of PD-1/PD-L1 coinhibitory signal is the expression of PD-L1 by nonhematopoietic cells, such as vascular endothelium (9). Although CTLA-4 signaling plays a predominant role in early T cell activation in secondary lymphoid organs, PD-1 may have a dominant regulatory function in peripheral tissues, based on the local PD-L1 expression (27, 29). Supporting the role of PD-L1 on the allograft, the survival advantage was abrogated if PD-1 Tg mice were transplanted with BALB/c hearts that lacked PD-L1. This finding reinforces prior observations suggesting a protective effect of overexpression of PD-L1 on donor grafts (30) and the deleterious effect of the absence of PD-L1 in either hematopoietic or nonhematopoietic cells in the graft in a chimeric PD-L1 mouse model (9). However, other reports show that allografts lacking PD-L1 are still accepted in mice treated with repetitive doses of CTLA-4–Ig but exhibited severe chronic rejection and vasculopathy (7). Our histopathology analysis of the BALB/c and bm12 grafts at 100 days and 8 weeks after the transplant, respectively, support the critical role of PD-1/PD-L1 signaling in preventing chronic rejection. In sum, PD-1/PD-L1 signaling in the graft seems to play a major protective role in the allograft.

We provide evidence that despite the decrease in Treg numbers, Tregs were required for the long-term graft acceptance in PD-1 Tg recipients. Known to regulate both central and peripheral tolerance, PD-1 is highly expressed on regulatory T cells and is involved in inducible Treg generation (16).We have demonstrated an increase in IL-10 and TGF-β signaling in PD-1 Tg Tregs in our transplant model, both of which are important pathways for Treg suppression. We also have shown that, compared with WT T cells, PD-1 Tg effector T cells proliferate significantly less in vitro and in vivo when exposed to alloantigens, and their cytokine secretion is blunted when stimulated ex vivo. This is in agreement with reports showing that PD-1/PD-L1 interaction results in inhibition of TCR-mediated lymphocyte proliferation (31, 32). Our findings also consolidate data showing that PD-1 activation in a fully MHC-mismatched cardiac transplant in mice results in the reduction of intragraft cytokine secretion such as IFN-γ (6).

Although STAT5 is known to support Treg function (21, 23), we observed that PD-1 overexpression decreased the activation of STAT5 upon IL-2 stimulation. Our data suggest that alternative intracellular molecular mechanisms exist by which Tregs can exert their inhibitory effects. Indeed, our data show that Tregs overexpressing PD-1 have a higher expression of coinhibitory molecules upon transplantation. We found that PD-1 Tg Tregs exhibited upregulation of ICOS both at mRNA and protein levels. Also, all graft-infiltrating Tregs from PD-1 Tg recipients were ICOS^+^. ICOS has a key role in controlling effector functions and survival of Tregs in models of oral (33) and respiratory tolerance (34), as well as type 1 diabetes (35). However, it was reported to be dispensable for the in vitro generation of Tregs from CD4^+^CD25^–^ cells (36). ICOS was also reported to be a marker for highly suppressive, differentiated, antigen-specific Tregs that can inhibit CD8^+^ T cell responses (37). Moreover, ICOS^hi^ Tregs are accumulated in the microenvironment of different tumors (38–40) and present a higher suppressive ability than ICOS^lo^ Tregs (40). In humans, Ito et al. (41) identified a population of thymic-derived ICOS^+^ Tregs that overexpressed CTLA-4 and produced high amounts of IL-10. The authors also found that CD28 engagement strongly inhibited the in vitro proliferation of ICOS^+^ Tregs. This suggests that in our model, early costimulation blockade in PD-1 Tg recipients would favor the proliferation and suppressive effects of ICOS^+^-expressing Tregs.

The costimulatory molecule ICOS has also been reported to play a critical role in effector T cell activation and differentiation. Therapies targeting the blockade of the ICOS/ICOSL pathway have been used to treat rejection in mice (24, 42). However, these therapies have failed to improve cardiac graft survival in monkeys (43). We hypothesized that unexpected effects on ICOS blockade on highly suppressive ICOS^+^ Tregs could contribute to these disparate results. Indeed, anti-ICOS treatment negatively impacted the suppressive function of Tregs in PD1–Tg recipients, as evident by reduced production of IL-10 and TGF-β as well as by reduced suppressive capacity in vitro.

Anti–CTLA-4 (44), anti–PD-1 (45), and anti–PD-L1 (46) Abs and their combinations have reached clinical use and have proven effective for cancer immunotherapy. PD-1–pathway inhibitors are now FDA-approved for therapeutic use in more than 20 types of cancer. However, in regard to immunosuppression, CTLA-4 fusion proteins are clinically approved for use in rheumatoid arthritis (47) and kidney transplantation (48), and a potentially novel PD-1 agonist is being tested in humans in a phase 1 trial (ClinicalTrials.gov identifier NCT03337022).

Given the complexity and the myriad escape mechanisms that the immune system possesses, immunosuppression for solid organ transplantation continues to require a multipronged approach (49), and efforts to identify novel potential targets are essential. The preclinical data we present in this report suggest that the PD-L1/PD-1 pathway is a promising target in clinical transplantation and warrants further investigation, especially in synergy with costimulation blockade.

## Methods

### Mice.

WT C57BL/6J (B6; stock no: 000664), B6(C)-H2-Ab1^bm12^/KhEgJ (bm12; stock no: 001162), BALB/cJ (stock no: 000651), and C3H/HeJ (stock no: 000659) mice were purchased from The Jackson Laboratory. Foxp3-GFP mice were a gift from the Alexander Y. Rudensky laboratory (Memorial Sloan Kettering Cancer Center) (50). Descriptions of PD-1–KO (51), PD-L1–KO (29), and T cell–specific PD-1 Tg mice (12) have been published previously. Briefly, in the PD-1 Tg mice, PD-1 is overexpressed under the hCD2 promotor (12). PD-1 Tg mice were crossed with Foxp3-GFP animals in our animal facility. All mice were at 8–12 weeks of age and were harbored and used following the Harvard Medical School and National Institutes of Health guidelines.

### Mouse abdominal heart transplantation.

Heterotopic intraabdominal vascularized cardiac transplant was performed using microsurgical techniques, as described by Corry et al. (52). When indicated, recipient mice were injected i.p. with a single dose of human CTLA-4–Ig (5 mg/kg on day 2 after transplantation; Abatacept; Bristol Myers Squibb). Graft survival was assessed by palpation of the heartbeat. Rejection was determined by complete cessation of palpable heartbeat and was confirmed by direct visualization after laparotomy. Graft survival is shown as the MST in days. Anti-CD25 (PC-61.5.3, 100 μg; catalog BE0012; Bio X Cell) was administered i.p. on days –7 and –1 before transplantation. Anti-ICOS (clone 7E.17G9; catalog BE0059; Bio X Cell) or rat IgG2b isotype control (clone LTF-2; catalog BE0090; Bio X Cell) was administered i.p. on day 0 (375 μg), followed by 250 μg on days 2, 4, and 6 after transplantation.

### Mouse cervical heart transplantation.

Cervical heart transplantation was done by end-to-side anastomosis of donor ascending aorta to recipient carotid artery and end-to-side anastomosis of donor pulmonary artery to recipient external jugular vein. Rejection was determined by complete cessation of palpable heartbeat and was confirmed by direct visualization after laparotomy.

### Histopathology.

Cardiac graft samples from transplanted mice were harvested from both PD-1 Tg and WT groups at 14, 60, or 100 days after transplantation. Grafts were then fixed in 10% formalin, embedded in paraffin, transversely sectioned, and stained with H&E or elastin stain. Using the revised ISHLT classification (53), a transplant pathologist blinded to treatment graded the degree of rejection (from 0 to 3).

### In vivo proliferation analysis using a GVHD model.

WT or PD-1 Tg splenocytes were labeled with CFSE and adoptively transferred (~6 × 10^7^/mouse) into sublethally irradiated (1000 rad) BALB/c mice. Lymphocytes were harvested 3 days afterward, and proliferation was assessed by flow cytometry, using a FACS Canto II with FACS Diva software (both from BD Biosciences).

### Influenza infection.

WT or PD-1 Tg mice were infected intranasally with 0.3 LD_50_ influenza A strain PR8 (strain A/Puerto Rico/8/1934 H1N1; Charles River Laboratories). Weight loss was measured daily, and mice were euthanized when weight loss reached 20% of starting weight. Viral loads were determined from RNA isolated from mice lung homogenates using the Direct-zol RNA Miniprep kit (Zymo Research). RNA (40 ng) was reverse transcribed into cDNA using the iScript cDNA Synthesis Kit (Bio-Rad). In a final volume of 20 μL, cDNA was amplified for the acidic polymerase gene of influenza A/PR8 using the primers and probe: forward primer, 5′-CGGTCCAAATTCCTGCTGA-3′; reverse primer, 5′-CATTGGGTTCCTTCCATCCA-3′; probe, 5′-6-FAM-CCAAGTCATGAAGGAGAGGGAATACCGCT-3′. Quantitative PCR was performed with a QuantStudio 3 PCR system (Applied Biosystems). Relative mRNA levels were calculated using the comparative Ct method, using the housekeeping gene *Hprt* as an internal control. All the harvested lungs were weighted, and data were expressed per mg of tissue.

### Isolation of organs lymphocytes.

To isolate cells from the native hearts, cardiac allografts, spleens, and draining lymph nodes, organs were excised, minced, and digested with 500 U/mL collagenase (Roche) for 30 minutes at 37°C, followed by incubation with 0.1 M EDTA in PBS, pH 7.2, buffer for 5 minutes before final suspension in 5 mM EDTA, 1% FBS in PBS, pH 7.2. Isolated cells were then mechanically dissociated through a Falcon 70 μm cell strainer (Corning), and RBCs were lysed using hypotonic ACK buffer (Lonza).

### Cytokine measurement by Luminex assay.

Splenocytes harvested at 2 weeks after transplantation from recipient mice were restimulated ex vivo by irradiated donor-type splenocytes (0.5 × 10^6^ responder cells with 0.5 × 10^6^ irradiated donor splenocytes) for 48 hours. Cell-free supernatants were analyzed by a multiplexed cytokine bead–based immunoassay using a preconfigured 21-plex mouse cytokine detection kit (Millipore). All samples were tested in triplicate wells.

### ELISPOT assays.

Mouse IFN-γ and IL-10 ELISPOT assays were performed using kits from BD Biosciences according to the manufacturer’s protocol. Briefly, 0.45 μm hydrophobic, high-protein-binding Immobilon-P membrane plates (Millipore) were coated with either IFN-γ or IL-10 capture Ab at 4°C overnight, followed by blocking with 10% FBS-supplemented RPMI1640 for 1 hour at room temperature. Lymphocytes were isolated from transplanted mouse spleens at day 21 after transplant by magnetic separation using the EasySep Mouse T Cell Isolation Kit (StemCell Technologies). We incubated 3 × 10^5^ T cells with 5 × 10^5^ irradiated donor (BALB/c), host-derived (B6), or third-party (C3H) splenocytes at 37°C and 5% CO_2_ for 24 hours for IFN-γ or 48 hours for IL-10 detection. Spots were detected and counted using an ImmunoSpot analyzer (Cellular Technology).

### Flow cytometry and cell sorting.

Cell suspensions were Fc-blocked for 20 minutes before staining for surface markers for 30 minutes in FACS buffer (2% rat serum in PBS) on ice. Cells were stained with fluorochrome-labeled mAbs against B220 (RA3-6B2), CD62 ligand (CD62L, MEL-14), CD44 (IM7), CD25 (PC61), and CTLA-4 (UC10-4F10-11) from BD Biosciences; CD4 (GK1.5), CD8 (53.6.7), TIM-3 (RMT3-23), and PD-1 (RMP1-30) from eBioscience; anti–phospho-S6 (Ser235/236) and phospho-Stat5 (Tyr694) were from Cell Signaling; and Abs against CD19 (6D5), LAG-3 (C9B7W), CD28 (37.51), ICOS (C398.4A), CD69 (H1.2F3), Ki-67 (16A8), Helios (22F6), LAP (TW7-16B4), and IL-10 (JES5-16E3) were from BioLegend. Intracellular Foxp3 staining was performed after treating cells with the eBioscience Foxp3 Fixation/Permeabilization solution. The staining of IL-10 was performed using the Fixation/Permeabilization Kit (eBioscience). For intracellular cytokine staining, cell suspensions were preincubated for 6 hours with 50 ng/mL PMA, 500 ng/mL ionomycin, and BD GolgiStop (BD Biosciences) in 10% FBS RPMI before Fc blocking, followed by surface staining, permeabilization, and intracellular staining of IL-10 at room temperature for 30 minutes. Unstimulated cells and an isotype control were used as controls. Cell viability was assessed using Fixable Viability Dye eFluo450 (eBioscience). A complete list of the Abs used can be found in the Supplemental Methods. All flow cytometry analyses were performed with the BD FACSCanto II or BD LSRFortessa (both from BD Biosciences) with FACS Diva software (BD Biosciences). Data were analyzed using FlowJo software (version X; Tree Star).

Splenic mouse Tconv (CD4^+^Foxp3-GFP^–^) and Tregs (CD4^+^Foxp3-GFP^+^) were sorted from naive or heart-transplanted animals (at day 21 after transplant) using a FACSAria cell sorter.

### In vitro regulatory T cell suppression assay.

Splenocytes from heart-transplanted recipients were flow sorted at day 21 after transplantation, and CD4^+^Foxp3-GFP^+^ Tregs and CD4^+^Foxp3-GFP^–^ cells were isolated. Tconv (CD4+GFP.Foxp3^–^) from the WT recipients were stained with 1 μM Violet Proliferation Dye (VPD450; Thermo Fisher) for 25 minutes at 37°C, followed by a 5-minute recovery in complete media at 37°C. Cells were washed twice and cocultured with WT or PD-1 Tg Tregs (CD4^+^Foxp3-GFP^+^) from each group at different ratios in the presence of anti-mouse CD3/CD28–conjugated beads (Thermo Fisher). Cells were incubated at 37°C for 72 hours and stained with viability dye (7-aminoactinomycin D; BioLegend), and proliferation was analyzed by flow cytometry in a BD LSRFortessa with FACS Diva software (both from BD Biosciences). The percentage of suppression was determined by the following formula: (Tconv proliferation without Tregs –Tconv proliferation with Tregs)/Tconv proliferation without Tregs.

### PCR array.

We extracted total RNA from flow-sorted WT or PD-1 Tg CD4^+^Foxp3-GFP^+^ cells (naive animals) using the RNeasy Kit (Qiagen). Synthesis of cDNA was performing using the RT^2^ First Strand Kit (Qiagen), followed by a preamplification using RT^2^ PreAMP Pathway Primer Mix for the Mouse T Cell Anergy & Immune Tolerance panel (Qiagen). Real-time PCR array was performed using an RT^2^ SyBR Green qPCR Mastermix using an ABI 7500 FAST machine (Invitrogen). The data were analyzed using the GeneGlobe Data Analysis Center from Qiagen.

### Statistics.

Kaplan-Meier survival curves were constructed, and a log-rank test was used to compare the allograft survival between groups. For independent 2-group comparisons, we used the unpaired 2-tailed Student’s *t* test for analysis. Two-tailed *t* tests were corrected for multiple comparisons using the Holm-Sidak method. For multiple group comparisons, the 1-way ANOVA or 2-way ANOVA test was used to determine differences, depending on the number of comparison groups. Multiple comparisons between levels were checked with the Tukey post hoc test for 1-way ANOVA and Sidak test for a 2-way ANOVA. Differences were considered significant at *P* ≤ 0.05. Prism software was used for data analysis and drawing graphs (GraphPad Software, Inc.). Data represent mean ± SD with at least 3 samples per studied group for all experiments. To create the heatmap of the MFI of the markers analyzed by flow cytometry, we used the Morpheus matrix visualization and analysis tool (Broad Institute; https://software.broadinstitute.org/morpheus). MFI values were represented by using the minimum and maximum of each independent row of the heatmap.

### Study approval.

All experiments were approved by the Massachusetts General Brigham IACUC under protocol nos. 2016N000250, 2017N000218, and 2020N000125. All mice were at 8–12 weeks of age and were harbored and used following the Harvard Medical School and National Institutes of Health guidelines.

## Author contributions

TJB and LVR helped design the study, perform experiments, interpret the results, and write the manuscript. NM, ITL, RBG, KS, TS, and SO assisted in the experiments. SC and KL transplanted the animals. JD performed the Flu infection experiments. AMP and AHS provided PD-1 Tg, PD-1 KO and PD-L1 KO mice. PC, JA, PTS, AHS, PC, XCL, and NM helped interpret the results and with manuscript preparation and editing.

## Supplementary Material

Supplemental data

## Version 1 11/09/2021

In-Press Preview

## Version 2 12/22/2021

Electronic publication

## References

[1] Keir ME (2008). PD-1 and its ligands in tolerance and immunity. Annu Rev Immunol.

[2] Riella LV (2012). Role of the PD-1 pathway in the immune response. Am J Transplant.

[3] Murakami N, Riella LV (2014). Co-inhibitory pathways and their importance in immune regulation. Transplantation.

[4] Schildberg FA (2016). Coinhibitory pathways in the B7-CD28 ligand-receptor family. Immunity.

[5] Sharpe AH, Pauken KE (2018). The diverse functions of the PD1 inhibitory pathway. Nat Rev Immunol.

[6] Ozkaynak E (2002). Programmed death-1 targeting can promote allograft survival. J Immunol.

[7] Tanaka K (2007). PDL1 is required for peripheral transplantation tolerance and protection from chronic allograft rejection. J Immunol.

[8] Koga N (2004). Blockade of the interaction between PD-1 and PD-L1 accelerates graft arterial disease in cardiac allografts. Arter Thromb Vasc Biol.

[9] Riella LV (2011). Essential role of PDL1 expression on nonhematopoietic donor cells in acquired tolerance to vascularized cardiac allografts. Am J Transpl.

[10] Morita M (2010). PD-1/B7-H1 interaction contribute to the spontaneous acceptance of mouse liver allograft. Am J Transpl.

[11] Sandner SE (2005). Role of the programmed death-1 pathway in regulation of alloimmune responses in vivo. J Immunol.

[12] Keir ME (2005). Programmed death-1 (PD-1):PD-ligand 1 interactions inhibit TCR-mediated positive selection of thymocytes. J Immunol.

[13] Nishimura H (2000). Facilitation of beta selection and modification of positive selection in the thymus of PD-1-deficient mice. J Exp Med.

[14] Sayegh MH (2003). Allograft rejection in a new allospecific CD4+ TCR transgenic mouse. Am J Transpl.

[15] Riella LV (2013). Jagged2-signaling promotes IL-6-dependent transplant rejection. Eur J Immunol.

[16] Riella LV (2012). Deleterious effect of CTLA4-Ig on a treg-dependent transplant model. Am J Transplant.

[17] Boenisch O (2010). TIM-3: a novel regulatory molecule of alloimmune activation. J Immunol.

[18] Wherry EJ (2011). T cell exhaustion. Nat Immunol.

[19] Raffin C (2020). T_reg_ cell-based therapies: challenges and perspectives. Nat Rev Immunol.

[20] Chi H (2012). Regulation and function of mTOR signalling in T cell fate decisions. Nat Rev Immunol.

[21] Mahmud SA (2013). Interleukin-2 and STAT5 in regulatory T cell development and function. JAKSTAT.

[22] Zheng Y (2007). A role for mammalian target of rapamycin in regulating T cell activation versus anergy. J Immunol.

[23] Burchill MA (2007). IL-2 receptor β-dependent STAT5 activation is required for the development of Foxp3+ regulatory T cells. J Immunol.

[24] Harada H (2003). The role of the ICOS-B7h T cell costimulatory pathway in transplantation immunity. J Clin Invest.

[25] Parry RV (2005). CTLA-4 and PD-1 receptors inhibit T-cell activation by distinct mechanisms. Mol Cell Biol.

[26] Fife BT (2009). Interactions between PD-1 and PD-L1 promote tolerance by blocking the TCR-induced stop signal. Nat Immunol.

[27] Okazaki T (2013). A rheostat for immune responses: the unique properties of PD-1 and their advantages for clinical application. Nat Immunol.

[28] Honda T (2014). Tuning of antigen sensitivity by T cell receptor-dependent negative feedback controls T cell effector function in inflamed tissues. Immunity.

[29] Keir ME (2006). Tissue expression of PD-L1 mediates peripheral T cell tolerance. J Exp Med.

[30] Dudler J (2006). Gene transfer of programmed death ligand-1.Ig prolongs cardiac allograft survival.. Transplantation.

[31] Freeman GJ (2000). Engagement of the PD-1 immunoinhibitory receptor by a novel B7 family member leads to negative regulation of lymphocyte activation. J Exp Med.

[32] Chinai JM (2015). New immunotherapies targeting the PD-1 pathway. Trends Pharmacol Sci.

[33] Miyamoto K (2005). The ICOS molecule plays a crucial role in the development of mucosal tolerance. J Immunol.

[34] Busse M (2012). ICOS mediates the generation and function of CD4+ CD25+ Foxp3+ regulatory T cells conveying respiratory tolerance. J Immunol.

[35] Kornete M (2012). ICOS-dependent homeostasis and function of Foxp3+ regulatory T cells in islets of nonobese diabetic mice. J Immunol.

[36] Guo F (2008). CD28 controls differentiation of regulatory T cells from naive CD4 T cells. J Immunol.

[37] Vocanson M (2010). Inducible costimulator (ICOS) is a marker for highly suppressive antigen-specific T cells sharing features of TH17/TH1 and regulatory T cells. J Allergy Clin Immunol.

[38] Miller AM (2006). CD4+ CD25 high T cells are enriched in the tumor and peripheral blood of prostate cancer patients. J Immunol.

[39] Strauss L (2008). Expression of ICOS on human melanoma-infiltrating CD4+ CD25 high Foxp3+ T regulatory cells: implications and impact on tumor-mediated immune suppression. J Immunol.

[40] Tu JF (2016). Regulatory T cells, especially ICOS^+^ FOXP3^+^ regulatory T cells, are increased in the hepatocellular carcinoma microenvironment and predict reduced survival. Sci Rep.

[41] Ito T (2008). Two functional subsets of FOXP3+ regulatory T cells in human thymus and periphery. Immunity.

[42] Schenk AD (2009). Effector functions of donor-reactive CD8 memory T cells are dependent on ICOS induced during division in cardiac grafts. Am J Transplant.

[43] Lo DJ (2015). A pilot trial targeting the ICOS–ICOS-L pathway in nonhuman primate kidney transplantation. Am J Transplant.

[44] Hodi FS (2010). Improved survival with ipilimumab in patients with metastatic melanoma. N Engl J Med.

[45] Larkin J (2015). Combined nivolumab and ipilimumab or monotherapy in untreated melanoma. N Engl J Med.

[46] Robert C (2015). Pembrolizumab versus ipilimumab in advanced melanoma. N Engl J Med.

[47] Kremer JM (2003). Treatment of rheumatoid arthritis by selective inhibition of T-cell activation with fusion protein CTLA4Ig. N Engl J Med.

[48] Vincenti F (2005). Costimulation blockade with belatacept in renal transplantation. N Engl J Med.

[49] Safa K (2013). Beyond calcineurin inhibitors: emerging agents in kidney transplantation. Curr Opin Nephrol Hypertens.

[50] Fontenot JD (2005). Regulatory T cell lineage specification by the forkhead transcription factor foxp3. Immunity.

[51] Keir ME (2007). PD-1 regulates self-reactive CD8+ T cell responses to antigen in lymph nodes and tissues. J Immunol.

[52] Corry RJ (1973). Heart transplantation in congenic strains of mice. Transpl Proc.

[53] Stewart S (2005). Revision of the 1990 working formulation for the standardization of nomenclature in the diagnosis of heart rejection. J Hear Lung Transpl.

