# Supplementary material for: Overexpression of PD-1 on T cells promotes tolerance in cardiac transplantation via ICOS-dependent mechanisms
Source: JCI Insight. 2021 Dec 22;6(24):e142909. doi: 10.1172/jci.insight.142909 (PMC8783692; doi:10.1172/jci.insight.142909)
Supplement: Supplemental data [file jciinsight-6-142909-s144.pdf]

Supplemental Data For

Overexpression of PD-1 on T cells Promotes Tolerance in Cardiac Transplantation via an ICOS-Dependent Mechanism

Thiago J. Borges,[1,2] Naoka Murakami,[2] Isadora T. Lape,[1,2] Rodrigo B. Gassen,[1] Kaifeng Liu,[1] Songjie Cai[2] Joe Daccache,[2] Kassem Safa,[3] Tetsunosuke Shimizu,[2] Shunsuke Ohori,[2] Alison M. Paterson,[4,5] Paolo Cravedi,[6] Jamil Azzi,[2] Peter T. Sage,[2] Arlene H. Sharpe,[4,5] Xian C. Li,[7] Leonardo V. Riella[1,2,3]

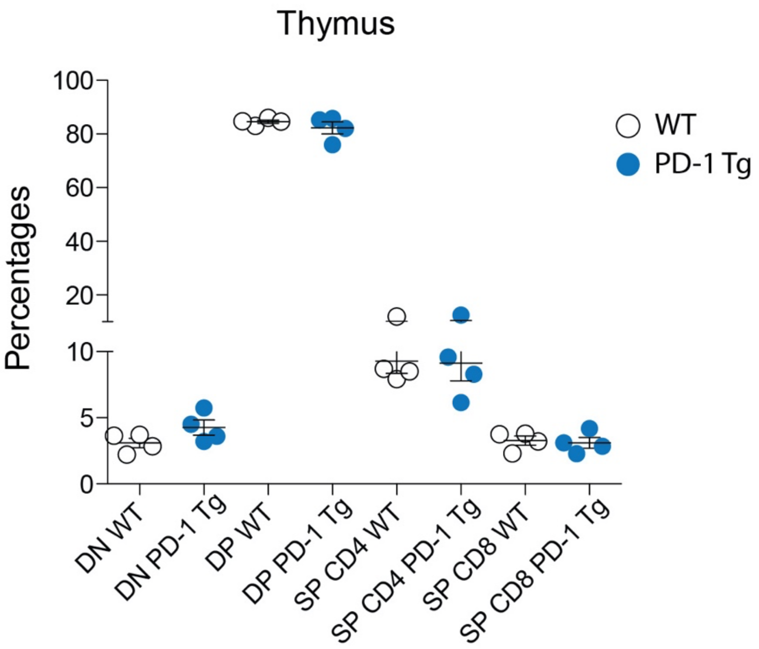

Supplemental Figure 1. Characterization of T cell development in the thymus. The average proportion of double-negative ($CD4^{-}CD8^{-}$), double-positive ($CD4^{+}CD8^{+}$) and single-positive cells ($CD4^{+}CD8^{-}$ or $CD4^{-}$ $CD8^{+}$) T cells in the thymus of WT and PD-1 Tg mice (n=4/group).

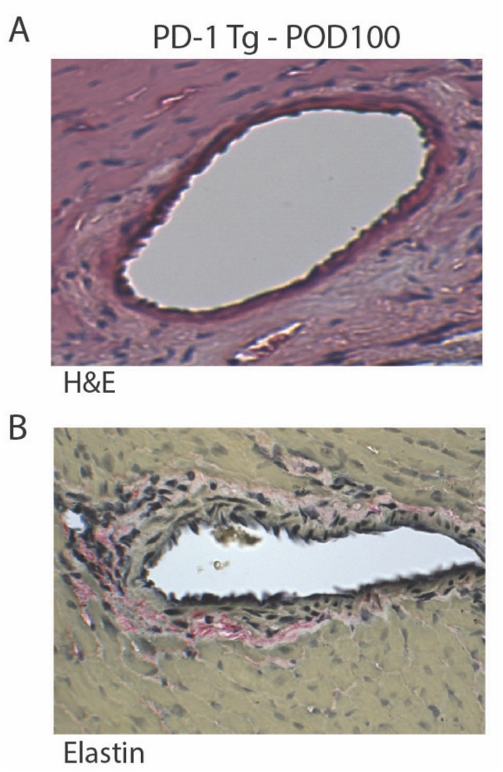

Supplemental Figure 2. Histopathology of the tolerized allografts in PD-1 Tg recipients. BALB/c hearts were transplanted into PD-1 Tg C57BL/6 mice with a single dose of CTLA-4-Ig (250 µg on day 2 post-transplant). Representative histology of cardiac allografts retrieved on day 100 post-transplant and stained with H&E and elastin (**A** and **B**, respectively) revealing a normal architecture, minimal lymphocytes infiltration and minimal intimal proliferation (n=6, 40x).

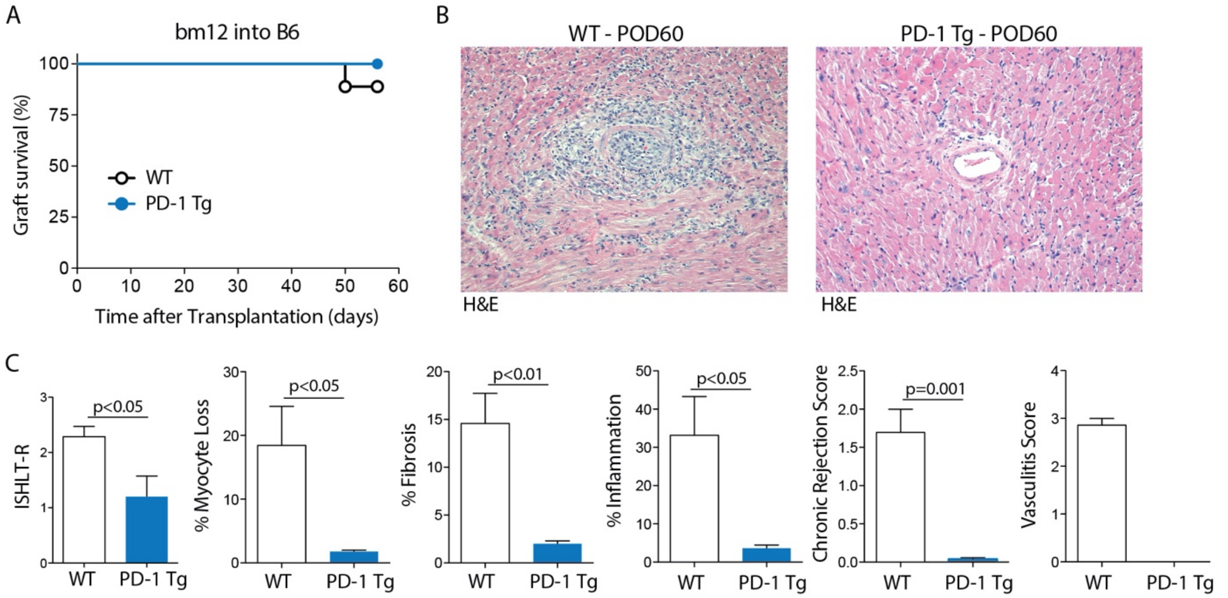

Supplemental Figure 3. Chronic rejection is less severe in PD-1 Tg mice compared with WT. (**A**) Kaplan-Meier curves of allograft survival: bm12 hearts were transplanted into PD-1 Tg or WT C57BL/6 recipients (n=7/group). (**B**) Representative histology of cardiac allografts retrieved on day 60 post-transplant and stained with H&E (20x). (**C**) Allograft histology at POD60 (~8 weeks) post-transplantation revealed significantly less rejection, inflammation, myocyte loss, fibrosis, chronic rejection and vasculitis scores in the PD-1 Tg group compared with WT. Statistics by t-test. The vasculitis scores in the PD-1 Tg group were zero for all recipients and statistical analyses were not applicable. ISHLT-R, consensus rejection score from the International Society of Heart and Lung Transplantation. For all panels, the bar graphs represent mean ± SD.

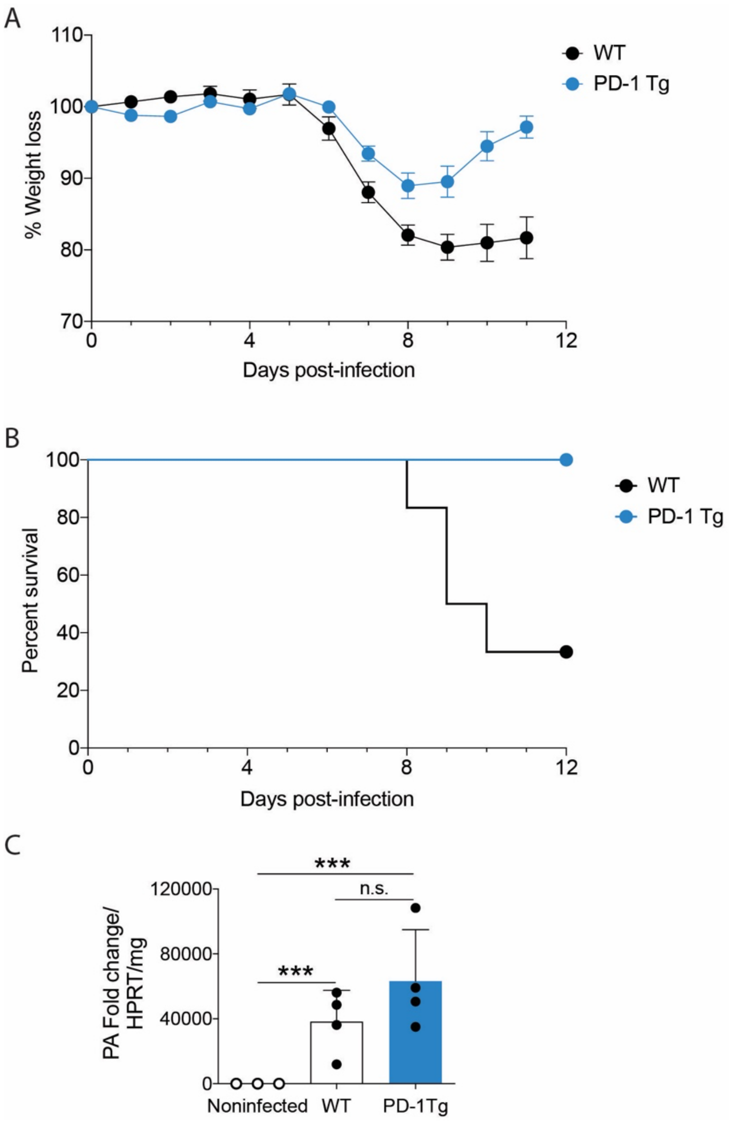

Supplemental Figure 4. PD-1 Tg animals are able to control Influenza infection. WT or PD-1 Tg animals were infected with 0.3LD$_{50}$ *Influenza* (strain A/Puerto Rico/8/1934 H1N1). Weight loss (**A**) and animal survival (**B**) were monitored daily until day 12 post-infection (n=6 animal per group). (**C**) Viral load of influenza A/PR8 acidic polymerase (PA) gene was determined in the lungs of WT or PD-1 Tg infected mice at day 7 post-infection. Data are represented as fold change from non-infected animals and were normalized by mg of the lung. The housekeeping gene *Hprt* was used as an endogenous control. All experiments are representative of two independent experiments. For all panels, the bar graphs represent mean ± SD.

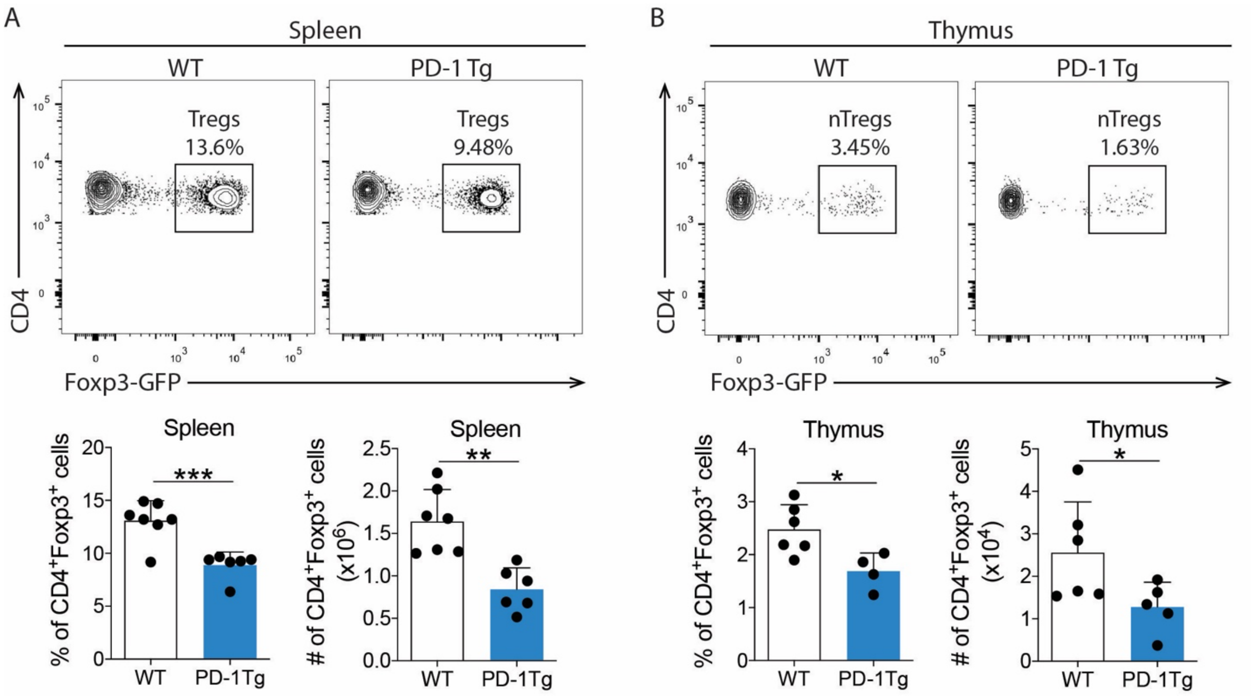

Supplemental Figure 5. Characterization of Tregs in spleen and thymus of WT and PD-1 Tg naïve animals. Average proportion and absolute numbers of Tregs ($CD4^+CD8^-Foxp3^+$) in (**A**) spleen and (**B**) thymus of WT and PD-1 Tg mice (n=4-7/group). Results are pooled from two experiments. For all panels, the bar graphs represent mean ± SD.

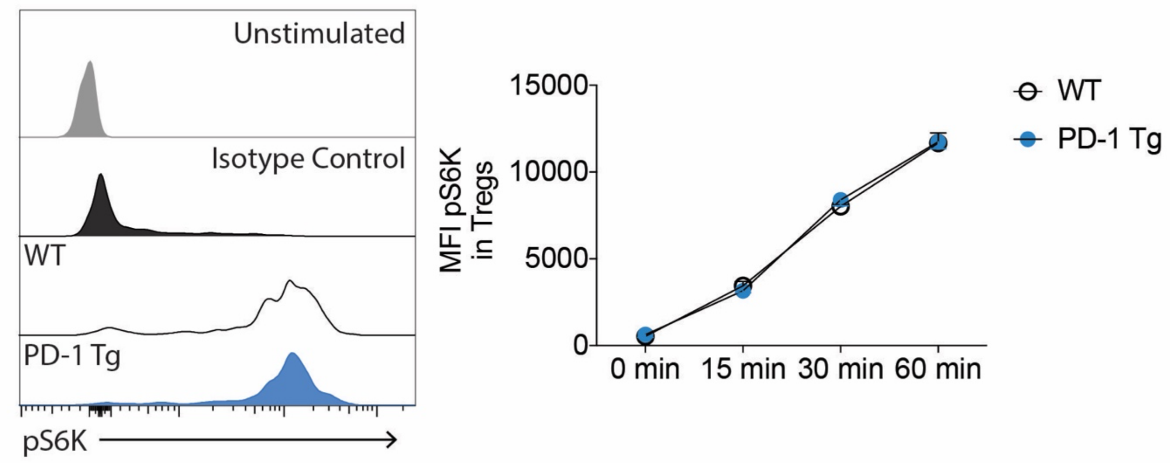

Supplemental Figure 6. Activation of the mTOR signaling pathway in WT or PD-1 Tg Tregs. Expression of phosphorylated-S6 kinase (pSS6K) in WT and PD-1 Tg Tregs stimulated with PMA/Ionomycin for 15, 30 or 60 min. Mean fluorescence intensity (MFI) was obtained by flow cytometry. Experiments were performed with a pool of three mice in triplicates. Statistic by two-way ANOVA with Sidak post-test.
